# Interactive extraction of diverse vocal units from a planar embedding without the need for prior sound segmentation

**DOI:** 10.3389/fbinf.2022.966066

**Published:** 2023-01-13

**Authors:** Corinna Lorenz, Xinyu Hao, Tomas Tomka, Linus Rüttimann, Richard H.R. Hahnloser

**Affiliations:** ^1^ Institute of Neuroinformatics and Neuroscience Center Zurich, University of Zurich and ETH Zurich, Zurich, Switzerland; ^2^ Université Paris-Saclay, CNRS, Institut des Neurosciences Paris-Saclay, Saclay, France; ^3^ Tianjin University, School of Electrical and Information Engineering, Tianjin, China

**Keywords:** acoustic signal processing, dimensionality reduction, segmentation problem, visual analytics, birdsong

## Abstract

Annotating and proofreading data sets of complex natural behaviors such as vocalizations are tedious tasks because instances of a given behavior need to be correctly segmented from background noise and must be classified with minimal false positive error rate. Low-dimensional embeddings have proven very useful for this task because they can provide a visual overview of a data set in which distinct behaviors appear in different clusters. However, low-dimensional embeddings introduce errors because they fail to preserve distances; and embeddings represent only objects of fixed dimensionality, which conflicts with vocalizations that have variable dimensions stemming from their variable durations. To mitigate these issues, we introduce a semi-supervised, analytical method for simultaneous segmentation and clustering of vocalizations. We define a given vocalization type by specifying pairs of high-density regions in the embedding plane of sound spectrograms, one region associated with vocalization onsets and the other with offsets. We demonstrate our two-neighborhood (2N) extraction method on the task of clustering adult zebra finch vocalizations embedded with UMAP. We show that 2N extraction allows the identification of short and long vocal renditions from continuous data streams without initially committing to a particular segmentation of the data. Also, 2N extraction achieves much lower false positive error rate than comparable approaches based on a single defining region. Along with our method, we present a graphical user interface (GUI) for visualizing and annotating data.

## 1 Introduction

Many real-world bioinformatics problems are best dealt with using semi-supervised approaches (Käll et al., 2007; Peikari et al., 2018; Wrede and Hellander, 2019), in particular when supervised and unsupervised methods are either unfeasible or unsuitable. For example, supervised learning is impractical when for a given task no training data is available or when the task definition is unclear, e.g., during explorative analysis. On the other extreme, unsupervised learning may provide interesting information, but perhaps not in a way that best suits a user, e.g., the components discovered in a data set may not be of the granularity of interest. The goal of semi-supervised learning is to combine the best of both supervised and unsupervised approaches to provide maximally useful insights with minimal human effort.

Animal behavior is an example domain well suited for semi-supervised learning, as each individual animal exhibits its own repertoire of complex actions. The task of segmenting behavioral sequences into their constituent parts is particularly challenging in the vocal space, because many vocal behaviors such as birdsongs consist of re-occurring elements that tend to be hierarchically organized (Sainburg et al., 2019) and that contain long-range structure (Markowitz et al., 2013). Here we set ourselves the goal of rapidly clustering the different types of vocalizations emitted by an individual songbird. For this type of problem, dimensionality reduction techniques come in handy because they allow to display even high-dimensional data points such as complex vocal utterances on a two-dimensional computer screen (Sainburg et al., 2019; Kollmorgen et al., 2020; Sainburg et al., 2020). However, in distance-preserving embeddings such as t-SNE (Maaten and Hinton, 2008) or UMAP (McInnes et al., 2018), the distance between two points in the plane only approximates the true distance between the pair of vocalizations in the higher-dimensional space of the original data (Kollmorgen et al., 2020). In fact, embedding distances are not perfectly preserved because local neighborhoods in two dimensions are much smaller than the true neighborhoods in the high-dimensional space. How to efficiently deal with such embedding distortions remains a bottleneck in data browsing, proofreading, and annotation tasks (Chari et al., 2021).

Moreover, natural vocalizations tend to have variable durations, which clashes with the rigid dimensionality of embeddings. Although there are workaround techniques such as zero padding, these depend on segmenting the signal into foreground and background as a preprocessing step, which tends to introduce errors caused by background noises. For example, when a background noise occurs just before or after a vocalization, that vocalization might be missed or inferred as being too long; and similarly, when a noise happens between two vocalizations, these might be interpreted as a single vocalization instead of as a pair. In general, to deal with the segmentation problem as a pre-processing step acts against end-to-end extraction of vocalizations from raw data. All these caveats and challenges limit the widespread adoption of dimensionality reduction techniques for annotating and proofreading vocalizations.

In general terms, the goal of our data annotation task is to extract flexibly defined and variably sized events from a continuous data stream. Our approach to vocal clustering is somewhat orthogonal to previous approaches where automated classifiers are optimized for the assignment of pre-computed segments to labels, either in a supervised (Tachibana et al., 2014; Nicholson, 2016; Goffinet et al., 2021; Steinfath et al., 2021; Cohen et al., 2022) or unsupervised manner (Sainburg et al., 2020; Goffinet et al., 2021). The goal there is to identify an efficient workflow that minimizes human involvement. In contrast, our aim is to place the human expert in the center of the process along the lines of modern visual analytics (Thomas and Cook, 2006; Cui, 2019). We want to provide the flexibility of exploring and navigating audio data while visually extracting diverse vocalizations in a fast and intuitive workflow.

We address the problem of clustering zebra finch vocalizations, which is to distinguish the diverse vocalizations they produce from all other sounds in the environment. In essence, the task is to correctly identify in midst of noise, all vocalizations including their types, their onset times, and their offset times. To robustly extract variable vocalizations from possibly distorted embeddings, we introduce for each vocalization type a pair of distinguishing feature sets, one anchored to the onset of the vocalization and the other to the offset. Hence, unlike traditional approaches, where vocalizations are represented by single dots in the embedding plane (Sainburg et al., 2019; Kollmorgen et al., 2020; Sainburg et al., 2020; Goffinet et al., 2021; Steinfath et al., 2021), in our workflow, vocalizations are represented by pairs of dots. Also, because we extract vocalizations without segmentation as a pre-processing step, our definition of vocalizations by their onset- and offset-anchored feature sets implicitly solves the segmentation problem.

## 2 Methods

### 2.1 Datasets and sound preprocessing

**FIGURE 1:**
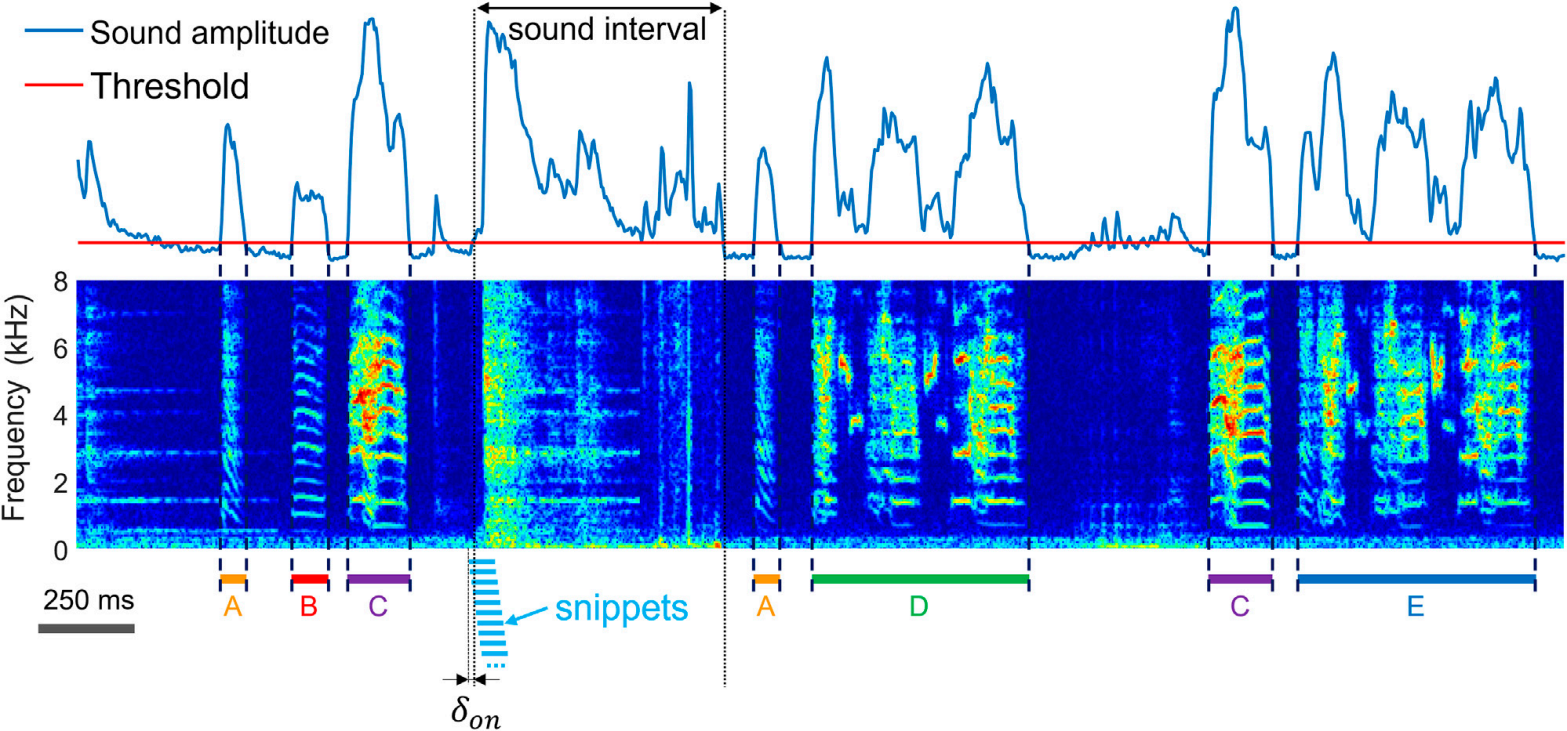
The task of extracting vocalizations is to correctly detect the onsets and offsets (dashed lines) of vocalizations and to determine their type **(A–E)** amidst diverse non-vocal sounds (noise). Shown is a time-frequency log spectrogram of adult zebra finch song. Our approach to extraction of vocalizations is to first extract sound intervals (based on threshold crossings of sound amplitude) and to dissect these into 64-ms long snippets (light blue bars) with 60 ms overlap among adjacent snippets. To achieve robustness to segmentation errors, we consider each snippet a potential onset or offset of a vocalization. The first snippet associated with an amplitude threshold crossing precedes the threshold crossing by a small margin $\delta_{on}$ (free parameter). The shown spectrogram was produced by concatenating two recording segments (at the second black dotted line), chosen to illustrate the diversity of vocalizations and noises.

Our data stem from single-housed birds (*n* = 2) recorded with wall-mounted microphones as described in Canopoli et al. (2014) or from a pair (*n* = 1) of birds which wore harnesses carrying accelerometers that signal body vibrations stemming from self-produced vocalizations (Anisimov et al., 2014). Although all birds have been recorded in acoustically isolated environments, the extraction of vocal units problem is in species such as the zebra finch that produce not just harmonic sounds but that also emit broadband vocalizations which can resemble non-vocal sounds (Figure 1; Supplementary Figure S1).

We sampled sound (and vibration) signals at 32 kHz and computed log-power spectrograms using the short-time Fourier transform in 512-sample Hamming windows and hop size among adjacent windows of 128 samples (i.e., 4 ms). We pre-segmented the data into sound intervals (assuming that without sound there is no vocalization) by thresholding the spectral power of microphone signals in the range 312 Hz to 8 kHz and of accelerometer signals (illustrated in Figure 3D) in the range 312 Hz to roughly 4 kHz. The threshold for sound interval extraction (Figure 1) was set to 5 standard deviations above the average spectral power calculated during periods of silence.

### 2.2 Neighborhood extraction from 2d-embedded spectrogram snippets

2N extraction was performed by dividing the spectrograms (within sound intervals) into spectrogram snippets of fixed width in the range of 12–16 columns (corresponding to snippet durations of 48–64 ms). The snippet duration is a variable that a user can adjust; in general, snippets should be large enough to yield robust separation of different vocalization types in the embedding plane and small enough to be able to cleanly slice a vocalization without cutting into adjacent vocalizations. The hop size between adjacent segments was given by one column (i.e., 4 ms), Figure 1. To the first data snippet associated with a sound interval, we ascribed a time lag =1 . The onset time $\delta_{on}$ of that snippet precedes the sound interval onset by $\delta_{on}=4$ spectrogram columns (i.e., 16 ms, Figure 1). Similarly, to the last snippet of a sound interval we associated a time lag =−1 ; the offset time of that snippet exceeds the sound interval offset by $\delta_{off}=6$ (i.e., 24 ms). These choices ensured that brief silent gaps before and after syllables were included in the defining characteristics of a vocalization. By definition, the second snippet of a sound interval had a time lag of d=2 (i.e., 4 ms) and the second-last snippet had a time lag of d=−2 (i.e., −4 ms), etc. This dissection of the data into snippets resulted in a total of 581k snippets for the day-long recording of the bird shown in Figure 1 (given the chosen snippet duration of 64 ms).

**FIGURE 2:**
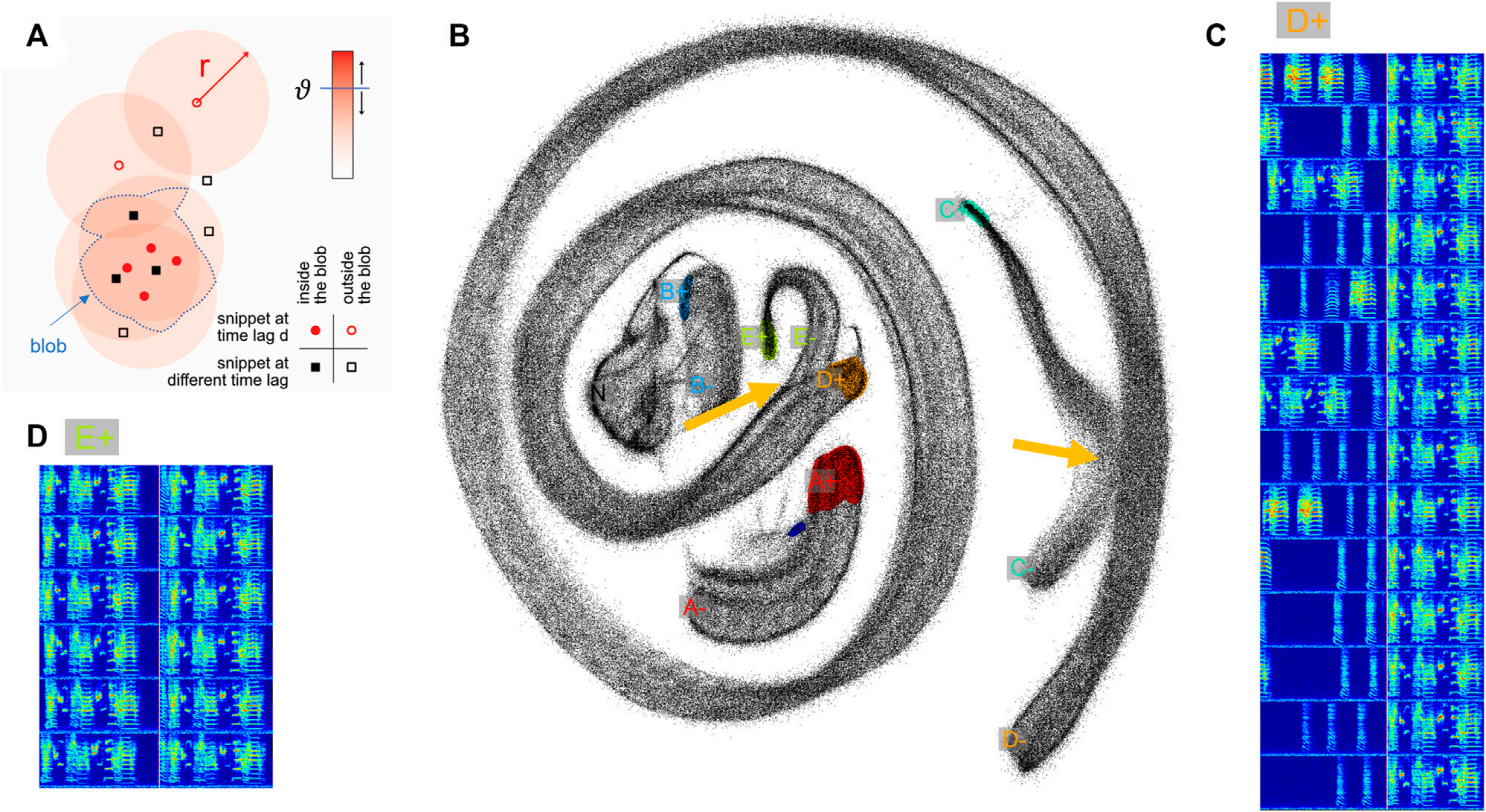
Two-neighborhood (2N) extraction of vocalizations from dense spectrogram embeddings. **(A)** Schematic illustrating the definition of a blob. After projecting all data snippets into the plane using UMAP, we replace the points corresponding to upward threshold crossings ( d=1 , red circles, first light blue bar in Figure 1) by large, filled disks (light red) of radius r that we sum up. All pixels at which the sum exceeds a given threshold ϑ (blue horizontal bar) are grouped into a blob (delimited by blue dashed line). All points that fall into this blob correspond to extracted vocalization onsets, including points that were dissected at a lag different from d (black filled squares). **(B)** Projected data snippets (black dots) from a one-day long recording. The onset blobs corresponding to the time slice d=1 are shown in color (different colors for different vocalization types). The yellow arrows point to indistinguishable snippet embeddings stemming from different vocalization types. The letters A-E indicate manually chosen onset slices “+” and offset slices “−” for each vocalization type (same lettering as in Figure 1). The cluster labelled ‘N’ (gray) is a noise cluster without distinct onset and offset behavior, this cluster was ignored. The small blue blob next to the A+ blob is an onset variant of the introductory note, which can either be included in the definition of A+ (introductory note) or excluded. **(C)**, **(D)** Spectrograms of example syllables taken from blob D+ **(C)** and blob E+ **(D)**. Same bird as in Figure 1.

We then embedded the snippets into the plane using UMAP (McInnes et al., 2018) and visualized high-density neighborhoods associated with a given time lag d, as follows: We drew a small disk of radius r around every point (Figure 2A, red dots) in the embedding plane that had a time lag of either d or d+1 if d was positive (onsets), and either d or d−1 if d was negative (offsets). Thresholding the number of overlapping disks per pixel with a density threshold ϑ revealed supra-threshold (high-density) regions of points, which we refer to as neighborhood-defining blobs (Figure 2A, outlined in blue). The blobs for d=1 are shown in Figure 2B.

**FIGURE 3:**
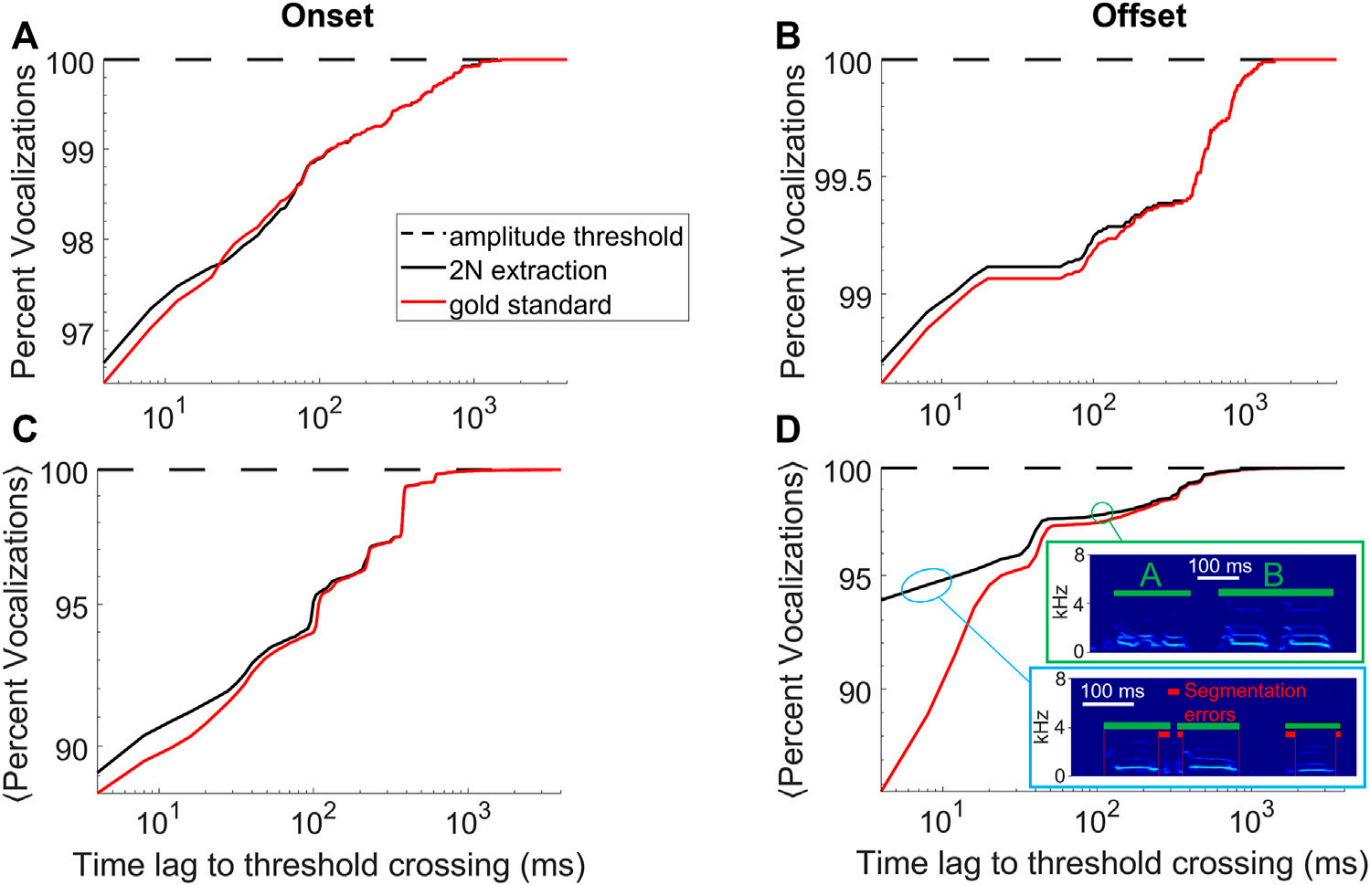
2N-extracted vocalizations (black curve) are similarly segmented as human-extracted vocalizations (red). **(A)** Shown is the cumulative percentage of vocalizations with an onset that falls within a given time lag (x-axis) following a sound amplitude threshold crossing. **(B)** Same for offsets that are within a given time lag preceding a sound-amplitude threshold crossing. Same bird as in Figure 1. **(C,D)** Cumulative percentage averaged across *n* = 4 birds. The insets in D show typical errors made by 2N extraction (green bars), which is to interpret a string of two calls as a single call (here labelled ‘B’, green bounding box) or to introduce segmentation errors (red bars) from too generous inclusion of surrounding noises (blue bounding box), data taken from an accelerometer-recorded bird. **(A–D)** Segments extracted by amplitude threshold crossings (dashed) trivially display no time lag whatsoever.

By changing the lag value d , blobs were interactively moved to unique regions along the 1d manifold of a vocalization type where no points from other vocalizations could be found (the latter we verified by visually inspecting spectrograms associated with a given neighborhood using our GUI). The points falling into a selected onset-related blob (Figure 2A, filled symbols) were then associated with that vocalization type. The vocalization onset times were given by the timestamp of the chosen snippets (i.e., the earliest snippet in case several adjacent points were found) minus the chosen time lag d (the smaller lag in case blobs were defined by two time slices). The time differences between the extracted vocalization onsets and the start times of the underlying sound intervals are shown as a cumulative density in Figure 3A. The analogous density for vocalization offsets relative to sound-interval endings is shown in Figure 3B and Figures 3C,D show the population averages.

#### 2.2.1 2N extraction algorithm

In the following, we describe the detailed extraction procedure of vocalizations, starting with their onsets. Extraction is parameterized by three variables: a time lag, a radius , and a density threshold ϑ . The 2N extraction algorithm consists of the following steps:

1) Define an integer time lag d>0 as small as possible (start with =1 ).
2) Identify all points in the embedding plane associated with this lag.
3) Replace each identified point with a disc of radius r and sum up these discs, yielding a 2d density.
4) Identify the regions where the 2d-density exceeds a threshold. These regions we refer to as blobs, they are the defining characteristics of vocalization onsets.
5) Change d, r, and ϑ to place and shape the blob such that it defines a uniquely characteristic region of the vocalization of interest. Ideally, choose d close to zero such that the blob is close to the onset.
6) Repeat steps 1–5 up to K times to define diverse onset blobs for a given vocalization type (typically, K=1 because there is a unique blob for each vocalization type).
7) Identify all points inside the K blobs. These points uniquely define the onsets of the extracted vocalizations given by their timestamps minus the anchoring time lag $d_{i}$ of the underlying blob ( i=1,…,K ).

In this procedure, the optimal choices of radius r and threshold ϑ depend on the local density of points and should be individually chosen for each vocalization type and each blob. Essentially, the radius should be chosen as large as possible to not miss any onsets and likewise the threshold should be as low as possible to make the blob as large as possible to maximize the number of points harvested. However, a blob should not extend into points associated with confounding vocalizations, so a bit of manual fine-tuning is required for each vocalization type.

When all onsets of a vocalization type are defined, we perform the analogous definition of offsets; the lags of offset-defining blobs satisfy <0 ; the parameters d, r, ϑ, and K of offset blobs needed similar fine-tuning in practice. Once both onset and offset blobs are defined for all vocalizations, we extract the vocalizations as the spectrogram regions delimited by an onset and a subsequent offset. Cases in which an onset was followed by an offset of another type were discarded. Also discarded were onsets following an onset with missing intervening offset (and *vice versa*, discarded were offsets following an offset with missing intervening onset). In the three birds analyzed, we extracted 5, 7, and 9 vocalization types per bird, respectively. We then visually inspected the extracted spectrograms and manually corrected segmentation and clustering errors. That is, onset and offset times were manually adjusted by an expert in 4-ms steps to the nearest true onset or offset; and, misclassified vocalizations were assigned to the correct vocalization type or to noise (we observed neither, see Section 2.3).

#### 2.2.2 Practical notes

In all adult zebra finches examined, we managed for each vocalization type to find distinct onset and offset blobs. We carefully selected each blob as close as possible to its extreme position, i.e., as close as possible to the onset resp. offset of the associated sound interval. In nearly all birds, we found at least one point cloud in the embedding plane corresponding to non-vocal noise; in this cloud it was impossible to select both onset and offset blobs, presumably because noise tends to be unstructured, i.e., for noise there were no distinct time lags to amplitude threshold crossings at which noise snippets appeared more similar with each other than with snippets at other lags. We therefore extracted noise segments as the time intervals from the extracted onset until the ending of the underlying sound interval or the next vocalization onset, whichever came first.

### 2.3 Evaluation measures

In all birds, all 2N-extracted vocalizations were correctly classified into their types, as revealed by visual inspection of spectrograms. However, we observed occasional segmentation errors, where syllable onsets or offsets slightly deviated from the assessment by an expert. To calculate the precision of 2N extraction, an expert determined for all 2N-extracted vocalizations of a given type the fraction f of correctly classified 4-ms time bins.

The goodness of the implicit segmentation obtained with 2N extraction was evaluated by comparing the onset times of the extracted sound intervals to the corresponding onset times of the manually curated vocalizations, shown as a cumulative density in Figure 3A. The same procedure was performed for offsets, shown in Figure 3B. Averages of onset and offset cumulative densities are shown in Figures 3C,D, the less deviation between 2N-extracted and human-annotated segments, the better the segmentation performance.

**FIGURE 4:**
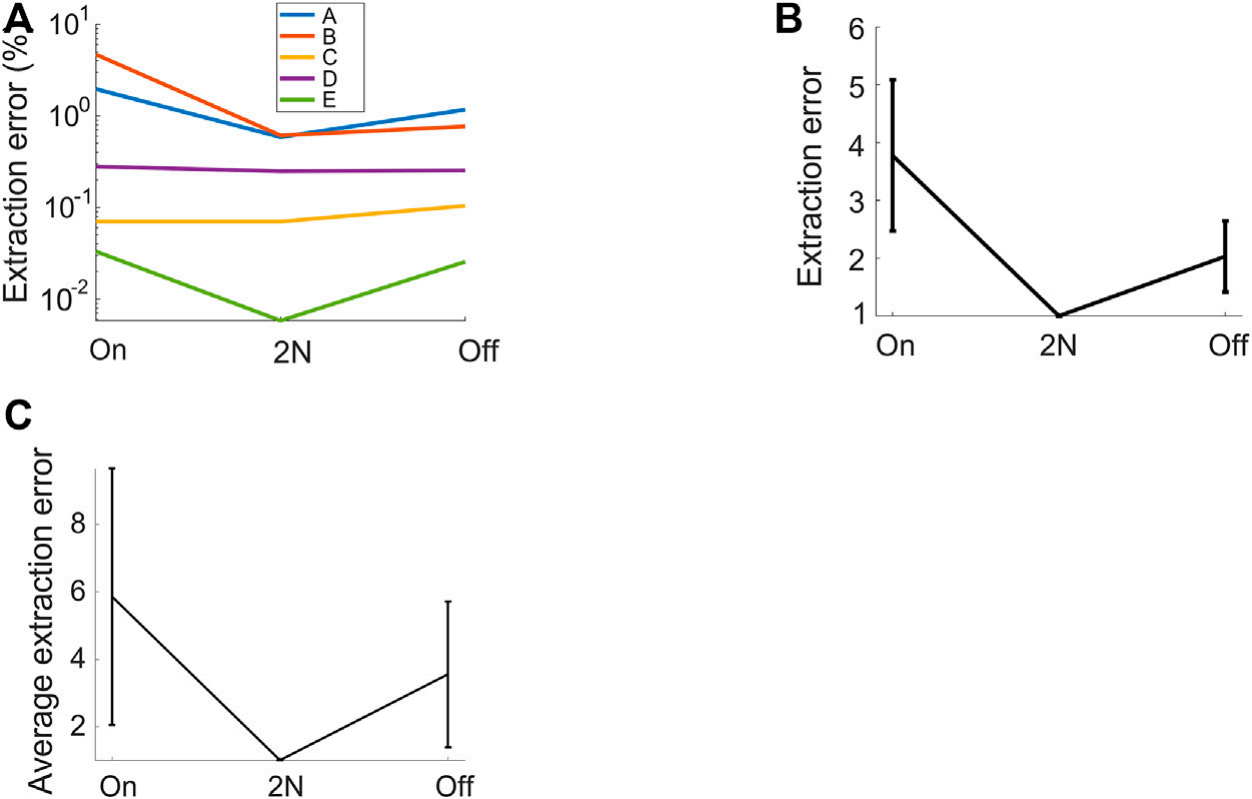
2N-extracted vocalizations achieve higher precision than their 1N-extracted counterparts. The fraction of extracted (4-ms) time bins that are misclassified (extraction errors) is shown for diverse methods: 2N extraction and either onset-anchored (on) or offset-anchored (off) 1N extraction. **(A)** For each vocalization type, the error is lower when vocalizations are extracted from two neighborhoods than when extracted from one neighborhood. Same bird as in Figures 1, 2. **(B)** Same data, averaged over all syllable types and after normalizing by the error rate of 2N extraction (shown is average ± std across vocalization types). The retrieval error of 1N extraction is 2–4 times higher than that of 2N extraction. **(C)** Normalized relative extraction error (average ± std across 4 birds). 2N extraction achieves 3–6 times fewer errors.

To quantify the benefits of defining vocalizations from both ends instead of just one, we repeated the same calculation for 1N-extracted vocalizations that were defined by considering only the onsets blobs or only the offset blobs rather than both. In the onset-anchored 1N baseline, we extracted a vocalization from the time difference $\Delta t=t_{a}-t_{b},$ where $t_{b}$ is the timestamp of a point in the onset blob minus the selected time lag d and $t_{a}$ is the end of the underlying sound interval or the timestamp of the next point in an onset blob, whichever came first. Analogously, in the 1N-offset baseline, we extracted a vocalization from the timestamp of a given point in the offset blob minus the selected (negative) time lag d , backwards, until the previous point in an offset blob or the beginning of the underlying sound interval, whichever came first (going backwards in time). The corresponding extraction errors ϵ=1−f are shown in Figure 4 for diverse vocalization types and birds.

## 3 Results

The first step of our extraction method is to detect sound intervals (as opposed to intervals of silence). In line with other approaches (SAP http://soundanalysispro.com, Avisoft http://www.avisoft.com), we assume that very often (but not necessarily always), a vocalization onset corresponds to a lower (low-to-high) threshold crossing of sound amplitude, and an offset corresponds to an upper (high-to-low) threshold crossing. In other words, we assume that either before or after a vocalization there are brief periods of silence, implying that sound intervals often begin and end with vocalizations. We then densely dissect the sound spectrograms associated with sound intervals into overlapping snippets (Figure 1). By considering each snippet as a potential vocalization onset or offset, the vocal segmentation problem remains unresolved at this processing step (it will be resolved at a later step).

We then project all spectrogram snippets into a plane, similar to the continuous UMAP embeddings in Sainburg et al. (2020). However, in our case, the first snippet of a sound intervals protrudes the sound-interval onset by $\delta_{on}$ and the last snippet protrudes the offset by $\delta_{off}$ (Figure 1), which makes vocalizations appear as distinct 1D structures in the embedding plane with clear boundaries rather than as a single excessively long 1d structure as in Sainburg et al. (2020). Provided that for a given vocalization type, there are sufficiently many vocal renditions that are precisely segmented by sound amplitude, the corresponding snippet embeddings will lie close to each other in the embedding plane and form a dense cloud of points. In general, we expect to find an extended cloud of points in the embedding plane for each vocalization type that ranges from self-similar onset snippets on one end to self-similar offset snippets on the other.

Among the cloud of onset-related points, we will also find points that display a nonzero lag to the nearest threshold crossing of sound amplitude. Namely, due to noise, we expect that some vocalizations are not cleanly segmented by sound amplitude but that instead are preceded or followed by a suprathreshold noise, which means that the time lag to an amplitude threshold crossing can be arbitrarily large. Such noisy vocalizations can nevertheless be correctly extracted with our method because for extraction we rely not on prior segmentation but on similarity with cleanly segmented renditions.

In short, to extract vocalizations of a given type, we define in the embedding plane a dense region of points (a blob) associated with the onsets of this vocalization type, and a blob associated with the offsets of that type. Blobs are defined by points of a given lag value *d*, see Figure 2A ( d=1 for onsets and d=−1 for offsets). To minimize effects of embedding distortions and to disambiguate confounding vocalization types, we set a blob’s lag variable to a suitable value. We set the size of a blob as a function of the local density of points by adjusting two parameters: a radius r and a threshold. The radius r sets the size of the disks that are placed at the locations of the embedding points and the threshold ϑ sets the height that the summed disks must exceed for a pixel to be included in a blob (Figure 2A), for details, see Methods Section 2.2.1.

The extracted vocalizations are then defined as the spectrogram chunks that start at the timestamp of a point in an onset blob minus the blob’s lag value d and end at the timestamp of the first subsequent point within an offset blob minus the blob’s lag value, i.e., we extract vocalizations simply within the shortest (lag-corrected) time intervals between pairs of points in an onset and an offset blob. Importantly, we harvest all points within a blob, including points that were sliced at different time lags than the blob defining lag. Therefore, although the definition of vocalizations depends on the time lags to amplitude threshold crossings, the harvesting is oblivious of these lags, and so our method can correctly extract vocalizations that are not cleanly segmented by sound amplitude.

When during this extraction process, an onset of a given type is followed not by an offset of the same type but by another type of event such as either an onset, the end of the sound interval, or the end of the file, we simply extract a vocalization from the onset until one time bin before the said event. Alternatively, when the onset is followed by an offset of another type, we extract no vocalization (to maximize precision^1^).

We illustrate our extraction method on a one-day-long recording of an isolated male zebra finch. We sliced the recorded sounds intervals into more than half a million snippets of 64 ms duration each that we embedded into the (2d) plane using UMAP. As can be seen in Figure 2B, snippets from different syllables can appear indistinguishable in the embedding plane, either 1) because a bird repeats an indistinguishable sub-syllable or note in a different context (i.e., as part of a different syllable as illustrated in Figure 2: parts of syllables E and D), or 2) because of UMAP projection errors (i.e., when nearest neighbors are hallucinated (Kollmorgen et al., 2020)—the latter we visually found to be quite common). When such non-discriminability occurs ateither an onset or an offset, the respective snippet loses its distinguishing characteristic for that syllable (Figure 2B, yellow arrows). This situation is quite common in zebra finches that tend to sing different syllable types with indistinguishable endings. This ambiguity implies that the spectrogram snippets near an upper threshold crossing do not uniquely define the ending of that syllable type (presumably the same is also true for some syllable onsets). As a workaround to such repetitive structure inherent in birdsong (and language for that sake), our method provides the freedom to define syllable endings and beginnings at fixed time lags away from threshold crossings, at places within a syllable where the defining snippet becomes unique for that syllable.

Using our GUI (Supplementary Material), users can increase and decrease the lag variable d to observe the blobs move around in the embedding plane until they reach a region in the plane where there are no confounding points from other vocalization types. Such confounding points can be recognized thanks to the elongated 1d-structure of vocalizations in the embedding plane (Sainburg et al., 2020): the confounding points are the ones where two different 1d-structures come too close to each other (see Figure 2B, yellow arrows). For example, the bird in Figure 2B produced two very long and complex song syllable types in rapid succession, whereby the second type (E) displayed an additional small down sweep at the syllable beginning, making it clearly distinct from Syllable D only by virtue of this down sweep (Figures 2C,D). As a result, the endings of syllables D and E in the embedding plane coincided with each other, which is why we had to define the offset-anchored blob E-not far from the onset-anchored blob E+ in a region where it was distinct from any neighborhood of D-, to make sure the endings of syllable D are not confounded with parts of syllable E.

For the bird shown in Figures 1, 2B, about 96.6% of extracted vocalizations had cleanly segmented onsets and about 98.7% had cleanly segmented offsets (i.e., onsets and offsets coincided with sound amplitude threshold crossings). In another bird, also recorded with a microphone and kept alone in a soundproof box, clean segmentation was even more frequent (99.9% for onsets and 99.0% for offsets, respectively). However, in birds housed in pairs and recorded with a wireless accelerometer mounted to their back, the fraction of cleanly segmented vocalizations was much lower (down to 72% for onsets and 89% for offsets, respectively). Thus, the level of noise depends strongly on the recording method, but our method allows harvesting vocalizations even in noisy situations.

### 3.1 Performance evaluation

How good are the extracted vocalizations? We computed two performance measures associated with the extraction procedure: 1) The *quality of the segmentation* in terms of the time differences of extracted onsets and offsets relative to gold standard human annotations; and 2) the *clustering performance* in terms of the false-positive error rate of misclassified time bins, again assessed by human experts (see Methods).

With regards to 1) the quality of the segmentation, for the bird shown in Figures 1, 2B and for both onsets and offsets, the time lags to threshold crossings were similarly distributed for 2N- and for human-extracted vocalizations (Figures 3A,B), suggesting that 2N extraction extracts vocalizations from background noise in similar manners as humans do. For both onsets and offsets in this bird, the largest time lags to amplitude-threshold crossings were up to 1 s long. In all birds tested (*n* = 2 mic and *n* = 2 accelerometer birds), we found similarity between 2N-extracted vocal segments and the human gold-standard counterparts, constituting a big improvement over simple sound amplitude thresholding (i.e., defining onsets and offsets as lower and upper crossings of sound amplitude thresholds, respectively, Figures 3C,D).

Some disagreements were seen when data was noisy; namely, we observed a tendency in human evaluators to segment the offsets earlier, which we found was often due to double misses that occurred in strings of calls where both an offset and the following onset were missed, leading to the hallucination of a much longer call than there actually was (Figure 3D). We do not evaluate workarounds for such problems but propose to fix them by detecting for each vocalization type the outlier renditions of excessively long durations and by discarding these. The shorter segmentation errors within 10–20 ms of true syllable offsets were almost always caused by inclusion of respiratory or movement artifacts near syllable boundaries (Figure 3D) and very rarely were they caused by truncations of parts of syllables, which would be more detrimental for subsequent feature-based syllable analysis. In summary, our method provides improved vocal segmentation compared to simple sound amplitude thresholding, in particular when recordings are noisy as in pair-housed birds recorded with animal-borne sensors.

With regards to 2) the clustering performance, an expert evaluated the precision of 2N extraction in terms of the fraction of 4-ms time bins that were assigned to the correct vocalization type. We were particularly interested in comparing our findings to a baseline of extracting vocalizations not from two defining (sets of) regions in the embedding plane, but from only a single region, either anchored to the onset or the offset, but not both. In these 1N extraction baselines, we extracted vocalizations from a point in a blob until either the next point in the blob or until the end of the sound interval, whichever came first (see Methods).

We found that 2N extraction outperformed 1N extractions by a large margin, achieving 3–6 times fewer extraction errors (Figure 4). The superior precision of 2N extraction came only at a minimal cost of lower recall. Namely, for the bird shown in Figure 1, 2N extraction retrieved almost as many time bins as did 1N extraction, namely 99.6%. On average (*n* = 4 birds), the fraction of time bins retrieved with 2N extraction was 96.9% relative to the mean number of bins retrieved with 1N extractions (averages across onset- and offset-based 1N extraction methods). Thus, the added benefit of much lower extraction error came only at a minimal cost of potentially retrieving fewer vocalizations. Thus, in terms of extraction performance, it pays off to extract vocal units in terms of two sets of defining characteristics, one anchored to the onset and the other to the offset. In terms of manual processing time, 2N extraction comes at the obvious cost of twice the workload compared to 1N extraction. However, given that 2N extraction can be routinely done within less than five minutes for an experienced user, irrespective of the size of the data set, this overhead seems negligible in practice.

## 4 Discussion

We presented a simple and intuitive method for extracting arbitrary vocal units in embeddings of a continuous stream of data. Embedding methods such as UMAP and t-SNE have been criticized for the distortions they can create, especially in genomic data (Chari et al., 2021). While we find similar distortions in vocal data, our workaround is to flexibly define vocal units based on regions in the embedding plane that are far from ambiguities and presumably also from distortions.

Our key contribution is to identify vocalizations *via* two sets of characteristics near the onsets and offsets, rather than through a single set of characteristics tied to either the onset, the offset, or even the entire vocalization. The benefits of this dual recognition are better segmentation and higher clustering performance, because 2N extraction is designed to suppress errors resulting from vocal ambiguities, cage noises, and embedding distortions. The method works best when onset and offset defining blobs are sufficiently far apart such that there is no overlap between them.

2N extraction is flexible and can be tailored to meet specific user requirements such as correctly detecting syllable variants even when they are composed of sub-syllables forming a small unwanted silent gap, which birds sometimes produce. Short gaps can be ignored for example by smoothing sound amplitudes before computing sound intervals (which our GUI allows). With smoothed amplitudes, split syllables are robustly extracted when the defining neighborhoods are tied to the stable sub-syllable parts (rather than the gap).

2N extraction makes most sense on large data sets because there is only a time penalty for computing the embedding but virtually no overhead for defining the onset and offset blobs. We routinely calculated UMAP embeddings of up to 1 million sound snippets using standard desktop PCs with 32 GB of RAM. Since our method is not tied to a particular embedding method, we expect it to work also on other planar embedding types (we obtained similar results on t-SNE (Maaten and Hinton, 2008) embedded data).

2N extraction is a very flexible annotation method, providing 4 + 4 degrees of freedom for each vocalization type: the radius, the threshold, the lag, and the number of blobs (for each onset and offset). As noted, the defining regions of a vocalization type need not constitute a connected set. For example, we could have chosen to combine syllables D and E in Figure 1 into a single syllable type by defining its onset characteristic in terms of the two blobs labeled D+ and E+ in Figure 2. Thus, our GUI provides the user with high flexibility of defining vocal units, which minimizes the need for postprocessing including the correction of segmentation errors.

Our human-centered workflow is in line with other semi-supervised methods in bioinformatics (Wrede and Hellander, 2019) that focus on reducing knowledge-requiring and time-consuming algorithmic optimization. For the problem of extracting vocalizations, we see it as an advantage that users can resolve ambiguous situations by making an informed decision after exploring the full vocal repertoire (such as deciding whether some vocalizations belong to the same type or not as in Figure 1, syllables D and E). Such fine decisions are part of critical data assessment (Thomas and Cook, 2006; Cui, 2019) and are common when working with animal data.

Currently, our method requires a pre-segmentation into sound intervals, as otherwise we do not obtain the time lag d needed for defining blobs and for extracting vocal units. In data that is so noisy that there are barely any vocalizations that are cleanly segmented by sound amplitude, our method is not trivially applicable. We would recommend trying to use another sound feature than sound amplitude to obtain blobs as in Figure 2A: Provided the feature identically dissects a significant number of vocalizations, high-density regions of dots should emerge in the embedding plane, which would make our method applicable.

We imagine that our approach to extraction of vocalizations can generalize to biological and physical processes other than vocalizations. Namely, we believe that our approach will work well for the extraction of units in natural processes that contain rigid elements that sequentially unfold in variable sequences and at variable speeds. The duration of entities of interest should be typically longer than the snippet size. When overlapping data snippets from such processes are projected onto the plane, elongated structures will result, ideally displaying uniquely defining beginnings and endings as in Figure 2B. Our method might also work for spatial rather than temporal data, provided that the same requirement of repetitive sequential structures applies. We hope that our GUI can be of use to researchers wanting to adopt our methods for their work and as a basis for further developments.

## Data Availability

The original contributions presented in the study are included in the article/Supplementary Material, further inquiries can be directed to the corresponding author.
