# Supplementary figures and images for: Interactive extraction of diverse vocal units from a planar embedding without the need for prior sound segmentation
Source: Front Bioinform. 2023 Jan 13;2:966066. doi: 10.3389/fbinf.2022.966066 (PMC9880044; doi:10.3389/fbinf.2022.966066)

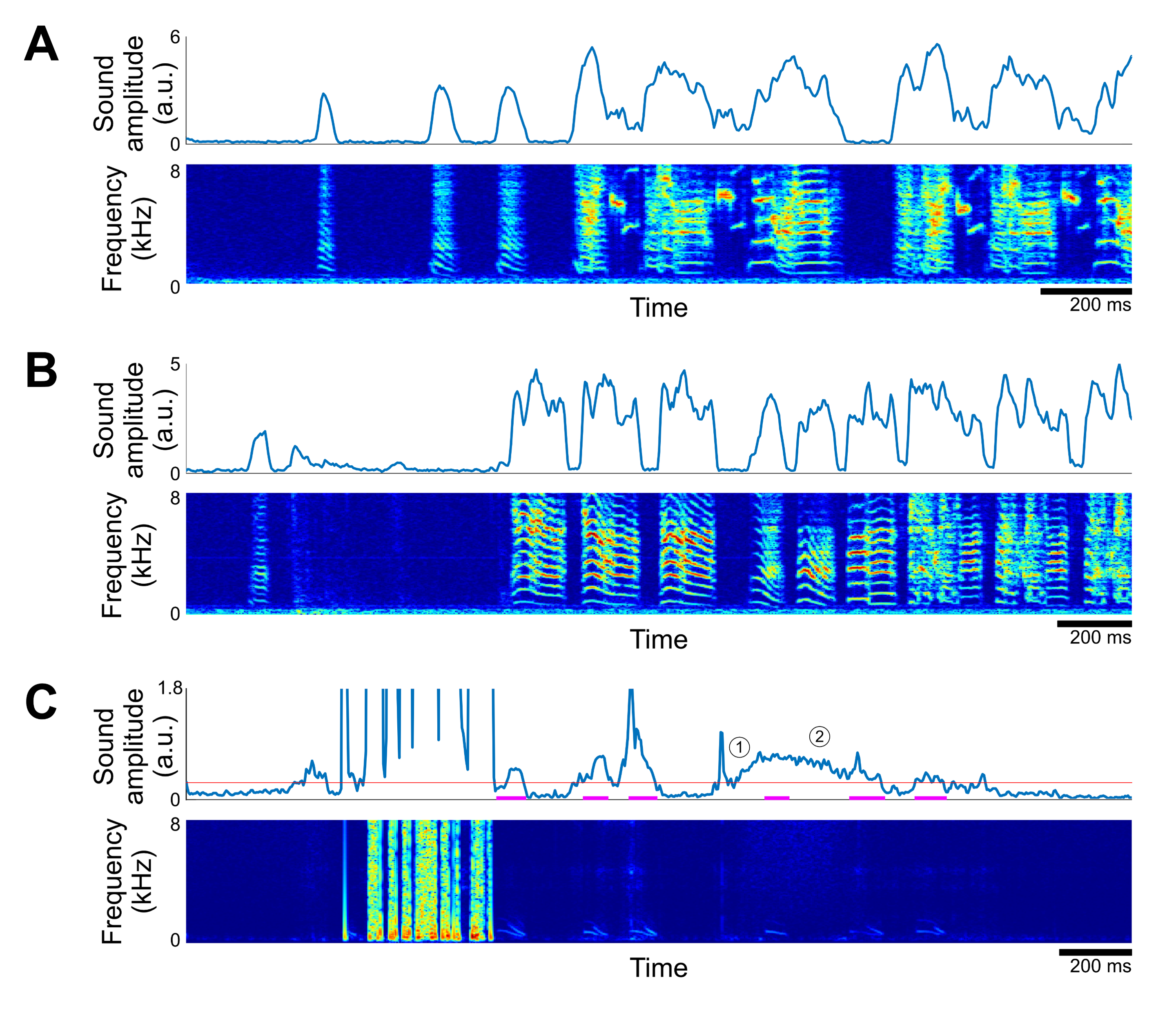

Supplement: Supplementary file 1 [file Image1.tif]
