# Supplementary material for: Interactive extraction of diverse vocal units from a planar embedding without the need for prior sound segmentation
Source: Front Bioinform. 2023 Jan 13;2:966066. doi: 10.3389/fbinf.2022.966066 (PMC9880044; doi:10.3389/fbinf.2022.966066)
Supplement: Supplementary file 2 [file DataSheet1.pdf]

A graphical user interface to extract extended vocal units from two neighborhoods in the embedding plane

In the main article, we propose a new approach to extract vocalizations from audio recordings which depends on user defined neighborhoods in a low-dimensional embedding of the continuously sliced audio signal. Here, we provide the GUI that we developed in parallel to the extraction strategy together with an example data set to illustrate the practical implementation of the approach.

Code, Data and Tutorials

The data set is a one-day recording of a single-housed male zebra finch and includes the spectrograms and the pre-computed UMAP embedding of the threshold-segmented and sliced audio signal. The repository with code, example data and tutorials can be found here on the ETH Research Collection or cloned from the https://gitlab.switch.ch/hahnloser-songbird/published-code/automtedclustering/2n-extraction.

Prerequisites

The GUI and auxiliary code were written in Matlab R2019b and require the Image Processing Toolbox and Curve Fitting Toolbox. The UMAP embedding requires the algorithm's implementation in Matlab from the file exchange server.

Practical User's Guide

To extract vocalizations from the example data, we recommend the following workflow

1. Execute *example_g17y2.m* which will load the data and open two figures (Figure 1):
   - The GUI (Matlab Figure 32) that visualizes the planar embedding
   - The context figure (Matlab Figure 24) that visualizes spectrogram snippets in their temporal context

2. In the GUI (Figure 1), you will see 't=1', which indicates the onset time slice. Pressing **right arrow** increases the time slice, which can be seen by the blobs moving around. Pressing **left arrow** decreases the time slice. Negative numbers indicate the time slice relative to the offset.

3. Mark both onsets and offsets of each vocalization type by **crtl + left mouse click** each on a positive and a negative time slice of that vocalization.[1] Half of the dots associated with that vocalization will turn pink. Choose your slice such that the blob is well distinct from other

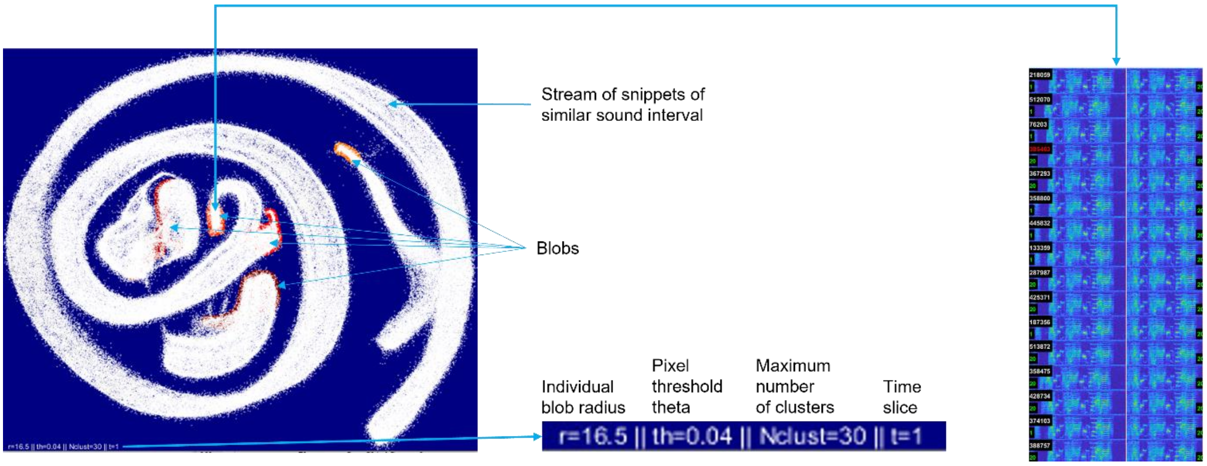

Figure 1. *The graphical user interface. Each snippet is a white dot in the planar UMAP embedding. Since subsequent snippets share a large fraction of overlap, similar sound events end up forming continuous streams. Users can browse through sound intervals and adapt the blob size to define selections and clusters of similar vocalizations.*

vocalizations. The true onset and offset are always back-calculated to 't=1' and 't=-1' respectively. The disk radius should be maximized to not miss any snippets outside the dense area, and the pixel threshold theta should be minimized to enlarge the blob. Vice versa, the blob should not be too large that it overlaps with confounding vocalizations. Press the **up** and **down arrow** to change the disk radius and **'–'** and **'='** to control theta.

Additional notes:

- Press the **spacebar** to change the visibility of the blobs and white dots.
- Press **shift + up arrow** and **shift++** to skip plotting (speed up your shaping of the blob).
- Press **0** to go back to the zero time slice.
- Press **shift + right arrow** or **shift + left arrow** to display the context of the current slice in Fig. 24 while you change time slice.
- When **crtl + left mouse click** on a blob, a figure will open showing the number of dots in different time slices covered by the currently selected blob.

4. When done clicking both an onset and an offset blob associated with a vocalization, press **w**. This will write the chosen onsets and offsets and the intermediate points to temporary variables. All purple dots will turn black.

5. If you have clicked the wrong blob or made some other mistake, click **c** to undo all control-clicks since last pressing 'w'. If you clicked 'w' wrongly, press **shift+c** to start again from scratch (point 3).

---

[1] Note that it is possible to define a cluster using just a single positive (but not negative) time slice. However, this is less precise than the two-neighborhood extracted vocalization and only advised to use for noise.

6. When all syllables are thus marked using ‘control-click’ and ‘w’, click **q** to finalize the extraction and close the GUI.

7. Inspect the extracted vocalizations in Fig. 24 pressing the **up and down arrow** to switch between vocalizations and the **mouse scroll wheel** to browse through the elements. Pressing ‘h’ lists more keypress functions that control the figure.

Terminology

Snippet A snippet is a very short window (64ms) of the spectrogram of s sound interval. As subsequent snippets are overlapping, they form trajectories in the embedding and snippets of similar vocalizations will be scattered along similar trajectories.

Disk A disk describes the circular kernel around a snippet. Increasing the disk radius increases the amount of overlap between nearby snippets and increases the chances of creating and enlarging a blob.

Theta The threshold theta defines how much disk overlap is required for creating blob. Changing theta will increase or decrease the blobs’ sizes.

Blob A blob is an area of overlapping disks where the number of overlaps is bigger than theta. Only snippets in the chosen time slice are considered for the blob.

Time slice The time slice reflects the position of the snippets within the sound interval. One can count forward, starting from the onset, or count backwards form the offset.

Cluster A cluster is a selection of similar sound events.

Keyboard shortcuts and mouse-controlled input

List of keyboard shortcuts

____________________DISK RADIUS

[↑, ↓] change the disk radius r by +/- 1

Shift + [↑, ↓] change the disk radius r by +/- 1 without plotting changes

____________________TIME SLICE

0 reset to time slice 0

[←,→] move forward or backward in time by +/- 1

Ctrl + [←,→] move forward or backward in time by +/- 10

____________________THETA

[-, =] decrease or increase theta by factor of 1.2

Shift + [-, =] decrease or increase theta by factor of 1.2 without plotting

Ctrl + [-, =] decrease or increase theta by factor of 1.02

____________________**MAX NUM OF CLUSTERS**

Ctrl + [↑, ↓]	change the maximum number of clusters by +/- 1

____________________**OTHERS**

delete	delete elements
w	assign current selection to cluster and inactivate the corresponding snippets
[1:9]	assign current selection to cluster 1:9
Shift + [11:119]	assign current selection to cluster 11:119
c	unselect last blob
shift + c	reset all selections and clusters
q	finalize clustering and save results
space	switch between showing only snippets, only blobs and snippets and blobs

List of mouse controls

left click	show elements in this blob in the context figure
ctrl + left click	select blob
scroll	move forward or backward in time
